# Predictors of Length of Hospital Stay Among Under-Five Children with Clinical Pneumonia at a Rural Tertiary Hospital Setting in South Africa

**DOI:** 10.3390/pediatric18030067

**Published:** 2026-05-13

**Authors:** Sanelisiwe Rosemary Mkhize, Olufunmilayo Olukemi Akapo, Siyonela Mlonyeni, Mirabel Kah-Keh Nanjoh

**Affiliations:** 1 School of Public Health, Faculty of Medicine and Health Sciences, Walter Sisulu University, Sisson Street, Fort Gale, Mthatha 5100, South Africa; djlnga07@gmail.com (S.R.M.); smlonyeni@wsu.ac.za (S.M.); 2 School of Laboratory Medicine & Pathology, Faculty of Medicine and Health Sciences, Walter Sisulu University, Sisson Street, Fort Gale, Mthatha 5100, South Africa; oakapo@wsu.ac.za

**Keywords:** childhood clinical pneumonia, prolonged length of hospital stay, predictors of prolonged hospitalization, rural, South Africa

## Abstract

Background: Pneumonia of viral and polymicrobial origin predominates the pathological profile of clinical childhood pneumonia, with high admissions rates in recent times. Identifying factors associated with prolonged hospital stay may aid in developing risk reduction strategies for improved admission outcomes. Methods: A facility-based historical cross-sectional study was conducted with a random selection of 186 medical records from January 2020 to December 2024 of children aged 0 to 5 with clinical pneumonia at a tertiary Hospital in Mthatha. Results: Over the five-year study period, clinical pneumonia accounted for 10.4% (95% CI: 9.8–11.1%, n = 950/9098) of the total under-five admissions. The median age was 108.5 (interquartile range (IQR) = 48.0–345.5) days, mainly comprising males (51.1%) and infants (65.2%), with viral (91.6%) and mild (88.0%) forms of pneumonia. The median length of hospital stay was 5 (IQR = 3.3–8) days, and 91 (49.5%) of the children had a prolonged hospital stay. In modified Poisson regression, infants [(relative risk (RR) = 2.7, 95% confidence interval (CI): 1.6–4.3), *p* < 0.001]; neonates (RR = 2.3, 95% CI: 1.2–4.6, *p* = 0.013); bacterial pneumonia (RR = 1.7, 95% CI: 1.2–2.6, *p* = 0.007); being hypoxic (RR = 2.2, 95% CI: 1.3–3.6, *p* = 0.003); absence of other respiratory tract infections (RR = 1.6, 95% CI: 1.2–2.1, *p* = 0.003), incomplete vaccination (RR = 1.5, 95% CI: 1.0–2.2, *p* = 0.038), non-usage of herbal medications (RR = 1.7, 95% CI: 1.3–2.2, *p* < 0.001), difficult breathing (RR = 1.7, 95% CI: 1.1–2.6, *p* = 0.028), current breastfeeding (RR = 0.6, 95% CI: 0.4–1.0, *p* = 0.048), other morbidities (RR = 0.7, 95% CI: 0.5–0.9, *p* = 0.002) were associated with prolonged length of hospital stay. Conclusion: Stratification of under-five children at admission according to age, type of pneumonia, vaccination status, and presence of other morbidities are needed to enhance monitoring and timely medical interventions.

## 1. Introduction

Pneumonia remains a leading cause of illness and death among children under five years of age, particularly in low- and middle-income countries (LMICs), where health system limitations and social determinants worsen disease outcome [1,2,3,4,5]. Globally, pneumonia is responsible for an estimated 700,000 deaths each year, accounting for approximately 14% of all deaths among children. Despite advances in immunization, antimicrobial treatment, and nutritional interventions, the disease continues to disproportionately affect vulnerable populations in sub-Saharan Africa and South Asia, where limited access to timely diagnosis and care contributes to delayed recovery and prolonged hospitalization [6,7,8,9]. In South Africa, pneumonia remains one of the leading causes of pediatric hospital admissions, placing a substantial burden on tertiary healthcare facilities, particularly in rural provinces such as the Eastern Cape [10,11]. The predominance of viral and polymicrobial infections, together with incomplete vaccination coverage and coexisting conditions such as HIV exposure, malnutrition, and suboptimal breastfeeding practices, increases the risk of severe disease and prolonged hospital stay [12,13,14,15]. Importantly, prolonged hospitalization in children not only reflects disease severity but also contributes to increased healthcare costs, risk of nosocomial infections, and poorer long-term outcomes [16,17]. Recent evidence has identified several important predictors of prolonged hospital stay, including younger age, bacterial etiology, incomplete immunization, and the presence of comorbid conditions [18]. Evidence from rural South African settings remains limited, despite the likelihood that socioeconomic inequities and resource constraints shape disease trajectories and clinical outcomes in these contexts. Addressing this gap is essential to inform context-responsive clinical guidelines, strengthen pediatric case management, and support national strategies to reduce preventable mortality among children under five [19,20,21]. Against this background, we investigated predictors of prolonged hospital stay among children under five years of age admitted with clinical pneumonia to a rural tertiary hospital in Mthatha, Eastern Cape. By identifying high-risk groups and potentially modifiable factors, this study aims to generate actionable evidence to inform clinical decision-making, guide targeted interventions, and improve health system efficiency in resource-constrained settings.

### 1.1. Study Design and Study Setting

A retrospective, facility-based cross-sectional study was conducted using medical records of children admitted with clinical pneumonia to Nelson Mandela Central Hospital (NMCH), Mthatha, South Africa, between January 2020 and December 2024. This design was chosen to examine patterns of morbidity and mortality and to identify predictors of clinical outcomes over the five-year study period. Data were extracted from ward registers and individual case files from the neonatal and pediatric wards. NMCH is a tertiary referral hospital located in the King Sabata Dalindyebo Local Municipality of the OR Tambo District, Eastern Cape Province. As a regional referral center for both public and private health services, the hospital provides specialized pediatric care, including the management of childhood pneumonia.

### 1.2. Study Population and Sampling

#### 1.2.1. Target Population

The study population comprised children aged 0–5 years who were admitted with a clinical diagnosis of pneumonia, as documented in hospital records. This age range was selected because it reflects the pediatric admission criteria used at Nelson Mandela Central Hospital and is consistent with South African pediatric pneumonia and World Health Organization (WHO) and case definitions [22,23].

Eligible participants were children aged 0–5 years with a clinical diagnosis of pneumonia, irrespective of etiology, who resided within the OR Tambo District and were admitted between January 2020 and December 2024. Children older than 5 years, those with incomplete or missing medical records, those admitted primarily for conditions other than pneumonia (such as congenital heart disease or primary asthma), and readmissions for the same pneumonia episode within a short interval were excluded.

#### 1.2.2. Sample Size Estimation

Epidemiological Data Analysis Program (EPIDAT) version 3.1 was used to calculate the sample size based on a pneumonia prevalence of 11.6% in South African children [22]. At a 95% confidence level and absolute precision at 5%, with the in-built formula Z^2^ × p × (1 − p)/e^2^, a sample size of 158 was estimated to be absolute precision:

Z for a confidence level (α) of 95% = 1.96.

p the proportion (expressed as a decimal) = 11.6 (0.116).

e the margin of error = 5% = 0.05

= (1.96)^2^(0.116) (1 − 0.116)/(0.05)^2^

=157.6~158.

An additional 15% (24 files) were added to compensate for incomplete data, resulting in a final sample size of 182 medical records; however, a sample size of 184 was reached for the study.

#### 1.2.3 Sampling Technique

A simple random sampling approach was used to select eligible records. Each file was assigned a unique numeric code and entered into Microsoft Excel, after which randomization was performed using EPIDAT. The randomized list was then cross-checked in excel to identify the final sample of 184 records. Files that did not meet the eligibility criteria were replaced by the next randomly selected record. Of the 950 pneumonia admissions recorded during the study period, 184 cases were randomly selected for detailed demographic, clinical, and pathological assessment. All subsequent analyses, including those of disease severity, comorbidities, vaccination status, and predictors of prolonged hospitalization, were restricted to this sampled cohort (n = 184).

### 1.3. Data Collection and Measurement

#### 1.3.1. Data Collection Procedure

Following ethical approval, data extraction was undertaken within the hospital records unit. Eligible records were identified from nursing logs, retrieved, coded, and reviewed using a structured data collection form. Data were extracted from each file in a standardized manner, and all records were securely refiled after review. Comorbidities were defined as coexisting medical conditions present alongside the primary diagnosis of pneumonia, specifically HIV infection, asthma, and diarrhea, as these conditions are known to influence disease severity and clinical outcomes in pediatric populations. Comorbidities were restricted to HIV status, asthma, and diarrhea, as these conditions were consistently documented in pediatric admission records and are recognized as important modifiers of pneumonia outcomes in children. Other potentially relevant comorbidities, including congenital heart disease, tuberculosis, and malnutrition, were not systematically analyzed because of inconsistent documentation in the retrospective records. Antibiotic treatment data were also included in the analysis. Pneumonia severity was classified according to the attending clinician’s documentation, guided by World Health Organization (WHO) criteria and South African Thoracic Society/Department of Health guidelines, including features such as bronchial breathing, bilateral crepitations, bilateral consolidation on chest X-ray (CXR) radiography, and tachypnea [23]. Comorbidities, and clinical outcomes were also extracted and analyzed only for the sampled cohort (n = 184), ensuring consistency across descriptive and inferential analyses.

#### 1.3.2. Data Collection Instrument

The data collection instrument was a structured, investigator-designed worksheet that was reviewed and validated by departmental reviewers and supervisors. It captured demographic and clinical variables, including age, sex, weight, height, immunization status, nutritional status, and place of residence. Nutritional status was assessed from information documented in patient records according to World Health Organization (WHO) growth standard classifications (WHO Anthro version 3.2.2, Geneva, Switzerland, 2011) [23], including underweight, normal weight, overweight, and obesity, based on weight-for-age and weight-for-height z-scores where available. Eligible records were reviewed by the primary investigator using a structured, pretested data extraction worksheet. Extracted information was checked for completeness and internal consistency during data collection. Place of residence was defined according to the child’s recorded residential setting and categorized as rural village, peri-urban township, or urban area. Clinical variables included presenting symptoms, symptom onset, diagnostic modality, pneumonia type and severity, comorbidities such as HIV, asthma, and diarrhea, vitamin A supplementation, breastfeeding status, timing of complementary feeding, and treatment regimen. Outcome variables included discharge status (alive or deceased) and length of hospitalization. Inpatient hospital stay is the number of nights spent in the hospital, calculated from the day of admission to the day of discharge [24]. The expected hospital stay is 5 days for children with pneumonia and has been applied in previous empirical studies [25]; thus, any stay above 5 days was considered prolonged. Given the retrospective nature of the study, some variables were incompletely documented in patient records. Files with incomplete or missing medical records that did not meet inclusion criteria were excluded from the study. For included records, variables with partial missing data were analyzed using available case analysis, and the corresponding denominators were reported for variables not available for all participants. No imputation of missing data was undertaken. Data extraction was performed by a single investigator using a structured, pretested worksheet; therefore, duplicate extraction and inter-rater agreement statistics such as kappa were not applicable.

#### 1.3.3. Validity and Reliability

Face Validity: Verified by academic supervisors for content appropriateness and clarity. Content Validity: Assessed by departmental reviewers to ensure comprehensiveness. Reliability: Ensured through uniform use of the structured worksheet and cross-checking extracted data for internal consistency and accuracy.

#### 1.3.4. Confounding Variables

Potential confounders malnutrition and HIV status were considered based on literature. Although no mortality was observed in the study, modified Poisson regression was used to adjust for possible predictors of prolonged hospital stay. Stratification was not necessary in this instance.

#### 1.3.5. Pilot Study

A pilot test was conducted on 10 records, which were excluded from the main study. This informed tool refinement and ensured alignment with study objectives.

## 2. Statistical Analysis

All analyses were conducted using IBM SPSS Statistics version 29 (IBM SPSS, Armonk, NY, USA, 2023) [26]. Descriptive statistics were used to summarize categorical variables (frequencies and percentages) and continuous variables (medians and interquartile ranges) due to non-normal distributions. Multivariable Poisson regression within the General Linear Model (GLM) framework was used to identify predictors of prolonged hospitalization. Modified Poisson regression was selected over binomial regression because it is specifically suited for modelling discrete, non-negative, right-skewed count data such as length of hospital stay [27]. The independence-of-observations assumption was met, as each child under five contributed a single observation, with no clustering or repeated admissions related to the current clinical pneumonia episode. Modified Poisson regression with robust standard errors was applied to account for the moderate overdispersion observed in the data (mean = 10.6, SD = 31.0, variance = 961.5; deviance/df = 1.706). This model provided the best overall fit, with notably lower AIC and BIC values (AIC = 365,832; BIC = 421,684) compared with the negative binomial model (AIC = 436,350; BIC = 492,202). The omnibus test for model significance was reported at *p* < 0.005. Results are presented as risk ratios (RR) with corresponding 95% confidence intervals (CI). The statistical threshold for significance across all analyses was set at *p* < 0.05.

## 3. Results

### Sociodemographic Characteristics of the Study Participants

Over the five-year study period, clinical pneumonia accounted for 10.4% (95% CI: 9.8–11.1%, n = 950/9098) of the total under-five admissions. Pneumonia-related mortality rate was 0.6 percent (95% CI: 0.3–1.3%, n = 6/950). The median age was 108.5 (interquartile range (IQR) = 48.0–345.5) days, mainly comprising males (51.1%), infants (65.2%), with normal weight (73.4%) and mild (88.0%) forms of viral (91.6%) pneumonia type. The majority had up-to-date immunizations (64.7%) and were still receiving formula milk (35.9%) (Table 1).

**Figure 1:**
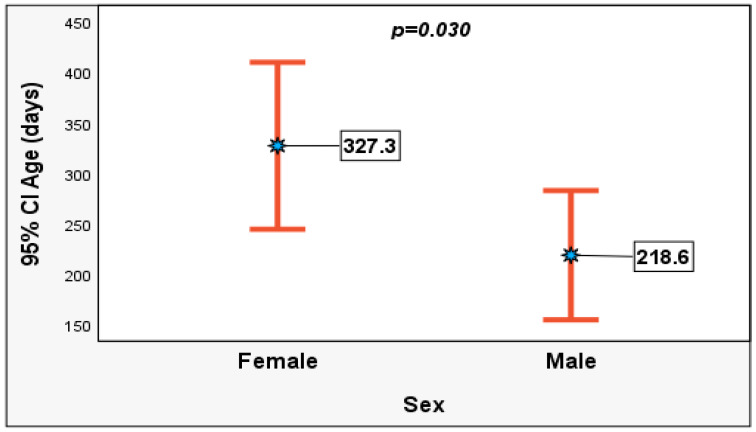
Error bar showing age difference between 184 male and female children under five admitted and treated for clinical pneumonia at Nelson Mandela Academic Hospital.

The female under-five children developed pneumonia at a statistically significantly older age (median = 177 IQR: 57–435 days; *p* = 0.030) than the male counterparts (median = 89 IQR: 43–271 days) (Figure 1).

## 4. Clinical Features at Presentation and Treatment Options

At presentation, classic symptoms including cough (82.6%) and hyperpyrexia (58.7%) were reported, and a diagnosis of pneumonia was established by chest radiographic examination in almost all (95.7%) of the children. The majority had features of difficulty breathing, including dyspnoea (59.8%), stridor (19.0%), wheezing (6.5%), and tachypnoea (4.9%). In addition, vomiting, diarrhoea, and loss of appetite were observed in 65 (35.3%), 33 (17.9%), and 23 (12.5%), respectively; and 63 (34.2%) were HIV exposed. Five different classes of pharmaceutical drugs, most commonly antibiotics (92.9%), were used for treatment, and one child required intermittent positive pressure ventilation. Other clinical features, history, and treatment options are presented in Table 2.

## 5. Length of Hospital Stay and Predictors

**Figure 2:**
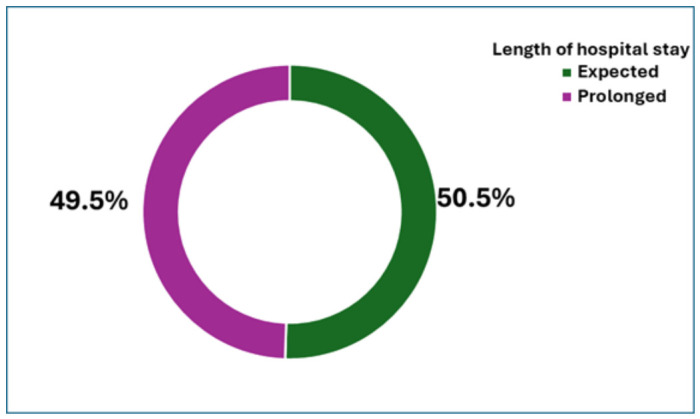
Length of hospital stay among 184 under-five children with pneumonia managed at a rural Eastern Cape, South Africa, referral hospital.

The median length of hospital stay was 5 (IQR = 3.3–8) days, and 91 (49.5%) of the children had a prolonged hospital stay (Figure 2).

Increased risk of prolonged hospitalization was observed among under-five children who were infants [(relative risk (RR) = 2.7 95% confidence interval (CI): 1.6–4.3), *p* < 0.001]; neonates (RR = 2.3, 95% CI: 1.2–4.6, *p* = 0.013); had bacterial form of pneumonia (RR = 1.7 95% CI: 1.2–2.6, *p* = 0.007); had hypoxia (RR = 2.2, 95% CI: 1.3–3.6, *p* = 0.003); did not have other respiratory tract infections (RR = 1.6, 95% CI: 1.2–2.1, *p* = 0.003), had not completed schedule vaccines for their age (RR = 1.5, 95% CI: 1.0–2.2, *p* = 0.038), did not use herbal medications (RR = 1.7, 95% CI: 1.3–2.2, *p* < 0.001), and had symptoms of difficulty in breathing (RR = 1.7, 95% CI: 1.1–2.6, *p* = 0.028). A 40% reduced risk (RR = 0.6, 95% CI: 0.4–1.0, *p* = 0.048) and 30% reduced risk (RR = 0.7, 95% CI: 0.5–0.9, *p* = 0.002) were observed among breastfed children and those with other morbidities, respectively. No significant predictive risks were observed for any of the other variables, as presented in Table 3.

## 6. Discussion

This study investigated predictors of prolonged hospital stay among children under five years of age admitted with clinical pneumonia to a rural tertiary hospital in South Africa. Nearly half of the children experienced prolonged hospitalization, underscoring the substantial burden of pneumonia on pediatric health services in resource-constrained settings. These findings are consistent with global evidence showing that pneumonia remains a leading cause of morbidity and mortality among young children, particularly in sub-Saharan Africa [28,29,30,31,32]. Infants and neonates were significantly more likely than older children to experience prolonged hospitalization. This finding is consistent with previous evidence showing that younger children are particularly vulnerable to severe pneumonia because of immunologic immaturity, smaller airway caliber, and increased susceptibility to hypoxemia [33,34,35,36,37]. In similar studies from Zambia and Malawi, infants had two- to three-fold higher odds of prolonged hospitalization than toddlers and preschool-aged children, underscoring the importance of age-specific strategies in pneumonia management [38,39,40,41,42]. Children with bacterial pneumonia were at greater risk of prolonged hospitalization than those with viral pneumonia. This is biologically plausible, as bacterial infections often present with more severe clinical manifestations and may require longer courses of antibiotic therapy, thereby delaying recovery [43,44,45]. Furthermore, comorbid conditions, including HIV exposure and other concurrent illnesses, were associated with length of hospital stay. This finding is consistent with evidence from Uganda and South Africa showing that children with HIV exposure or malnutrition tend to experience poorer clinical outcomes and delayed recovery [46,47]. Our findings further suggest that, unexpectedly, HIV-unexposed children had a higher risk of prolonged hospitalization. This may reflect differences in health-seeking behavior, earlier initiation of antiretroviral therapy among HIV-exposed infants, or residual unmeasured confounding, and therefore warrants further investigation. Incomplete vaccination also emerged as an independent predictor of prolonged stay, reinforcing the protective role of immunization in reducing pneumonia severity and improving clinical outcomes. Consistent with our findings, studies from Kenya and Nigeria have reported that children with missed or incomplete vaccinations are at increased risk of severe pneumonia and delayed recovery [48,49,50]. A particularly important finding of this study was the significant association between hypoxia and prolonged hospital stay. Children who were hypoxic had more than twice the risk of extended hospitalization compared with those without hypoxia, identifying hypoxia as one of the strongest predictors in the model. Clinically, hypoxia reflects impaired gas exchange and more extensive pulmonary involvement, often signaling severe pneumonia, ventilation–perfusion mismatch, or impending respiratory compromise. Such children typically require oxygen therapy, closer monitoring, repeated clinical assessment, and, in some cases, escalation to advanced respiratory support, all of which may prolong recovery and delay discharge [51,52]. Recent evidence continues to identify hypoxaemia as a major predictor of poor outcomes in children with pneumonia, including increased mortality risk and greater need for inpatient care [47,53,54,55]. In contrast, current breastfeeding was associated with a lower risk of prolonged hospitalization, underscoring its protective immunological benefits through the transfer of maternal antibodies and enhanced nutritional resilience. This finding is consistent with recent systematic reviews identifying breastfeeding as a key protective factor against both the incidence and severity of pneumonia in low- and middle-income countries [56,57,58,59]. The high prevalence of prolonged hospitalizations in this rural setting underscores the dual burden of clinical severity and health system constraints. Extended hospital stays increase the risk of nosocomial infection, disrupt household economic stability, and place additional pressure on resource-limited healthcare systems [60]. Respiratory support was analyzed but was not a significant predictor in this study. Identifying children at risk at the point of admission may facilitate prioritized monitoring, timely interventions, and more efficient allocation of healthcare resources. Our findings suggest that simple admission-level factors may be useful for risk stratification among under-five children hospitalized with pneumonia in rural settings. Early identification of children at risk of prolonged stay could assist clinicians in prioritizing monitoring intensity, oxygen-related resources, and inpatient bed utilization in hospitals where pediatric resources are limited. Furthermore, strengthening vaccination coverage, scaling up breastfeeding promotion, and improving early diagnosis and management at primary care level could mitigate the burden of prolonged hospitalization.

## 7. Strengths and Limitations

A major strength of this study is the use of real-world hospital data spanning a five-year period in a rural setting, which enhances its relevance for policy and practice. However, the study is limited by its retrospective design and reliance on secondary data, both of which may be affected by incomplete clinical documentation. Data extraction was conducted by a single investigator; duplicate extraction and formal inter rater agreement testing were not performed, which may have increased the risk of measurement error despite the use of a structured and pilot-tested extraction tool. Antibiotic use was common in the cohort, the retrospective records did not consistently document the route, duration, or specific clinical justification for prolonged intravenous antibiotic therapy; therefore, its direct contribution to prolonged hospitalization could not be determined. Additionally, the study did not include long-term outcomes post-discharge, which are important in understanding the full burden of childhood pneumonia.

## 8. Conclusions

Our findings demonstrate that younger age, bacterial pneumonia, incomplete vaccination, and comorbidities are key predictors of prolonged hospital stay, while breastfeeding exerts a protective effect. These results emphasize the importance of strengthening preventive strategies, particularly immunization and infant feeding practices, alongside risk stratification by age, symptoms, pneumonia type, and immunization status at admission to optimize care delivery. Future prospective studies are needed to validate these predictors and inform targeted interventions for high-risk children in rural South Africa.

## Figures and Tables

**Table 1:** Sociodemographic characteristics of 184 under-five children with pneumonia treated at a referral hospital in rural Eastern Cape, South Africa.

Variables	Categories	Frequency (n) N = 184	Percent (%)
Sex	Female	90	48.9
	Male	94	51.1
Age groups	Infants	120	65.2
	Neonates	23	12.5
	Preschoolers	8	4.3
	Toddlers	33	17.9
Age (days)	Median (IQR)	108.5	48.0–345.5
Weight (Kg)	Median (IQR)	5.9	4.0–9.1
Nutrition status	Underweight	40	21.7
	Normal weight	135	73.4
	Overweight	9	4.9
Type of pneumonia	Bacterial	13	7.1
	Fungal	1	0.5
	Polymicrobial	2	1.1
	Viral	168	91.3
Severity	Mild	162	88.0
	Severe	22	12.0
Breastfeeding status			
	Exclusive breastfed	48	26.1
	Not exclusively breastfed	11	6.0
	Not breastfed	33	17.9
	Unspecified	92	50.0
Current feeding practices, n = 92			
	Breastfeeding	18	19.6
	Formula feeding	33	35.9
	Mixed feeding	11	12.0
	Solid foods	30	32.6
Vaccination status			
	Inappropriate	65	35.3
	Appropriate	119	64.7

Nutrition status based on Weight-for-age z-scores. Inappropriate vaccination status: partial, no vaccination, and unknown vaccination status.

**Table 2:** Clinical characteristics at presentation and treatment options of 184 under-five children with pneumonia at a referral hospital in rural Eastern Cape, South Africa.

Variables	Frequency (n) N = 184	Percent (%)
Chest radiographic examination performed		
Yes	176	95.7
No	8	4.3
Clinical features		
Cough	152	82.6
Dyspnoea	110	59.8
High fever (temperature > 38.5 °C)	108	58.7
Stridor	35	19.0
Wheezing	12	6.5
Irritability	12	6.5
Tachypnoea	9	4.9
Hypoxia	6	3.3
Other clinical features		
Vomiting	65	35.3
Diarrhoea	33	17.9
Loss of appetite	23	12.5
Bronchiolitis	19	10.3
Nasal congestion	16	8.7
Seizures	4	2.2
Night Sweat	4	2.2
Weight loss	2	1.1
Clinical history		
Other respiratory tract infections	15	8.2
Other morbidities	51	27.7
HIV exposed	63	34.2
ART initiated, n = 63	21	33.3
HIV positive, n = 63	6	9.5
Supplemented at home	13	7.1
Previous hospitalization	8	4.3
Use of herbal medication	7	3.8
Treatment		
Antibiotics	171	92.9
Corticosteroid	31	16.8
Bronchodilator	22	12.0
Antifungal	21	11.4
Antiviral	3	1.6
Management of respiratory symptoms		
Non-invasive respiratory support	42	22.8
Invasive respiratory support	1	0.5
Non-invasive respiratory support, n = 42		
Low-flow nasal cannula	36	19.6
High-flow nasal cannula	6	3.3

**Table 3:** Predictors of prolonged hospital stay among 184 under-five children with pneumonia managed at a referral hospital in rural Eastern Cape, South Africa.

Variables	Std. Error	Relative Risk (95% Confidence Interval)	*p*-Value
Sex			
Female	0.111	1.1 (0.9–1.4)	0.442
Male			
Age groups (years)			
Infants	0.250	2.7 (1.6–4.3)	<0.001
Neonates	0.342	2.3 (1.2–4.6)	0.013
Preschoolers	0.236	0.7 (0.5–1.1)	0.163
Toddlers		1	
Nutrition Status (WAZ)			
Underweight	0.337	1.5 (0.8–2.8)	0.257
Normal weight	0.227	1.1 (0.7–1.6)	0.826
Overweight		1	
Type of pneumonia			
Bacterial	0.208	1.7 (1.2–2.6)	0.007
Viral		1	
Severity			
Severe	0.280	1.6 (0.9–2.8)	0.086
Mild		1	
Bronchiolitis			
No	0.211	1.2 (0.8–1.9)	0.320
Yes		1	
Hypoxia			
Yes	0.261	2.2 (1.3–3.6)	0.003
No		1	
Other Respiratory tract infections			
No	0.153	1.6 (1.2–2.1)	0.003
Yes		1	
Incomplete vaccination status			
Yes	0.190	1.5 (1.0–2.2)	0.038
No		1	
Use of herbal medications			
No	0.123	1.7 (1.3–2.2)	<0.001
Yes		1	
Vomiting			
No	0.104	1.0 (0.8–1.2)	0.966
Yes		1	
Diarrhoea			
Yes	0.180	1.0 (0.7–1.4)	0.996
No		1	
Seizures			
No	0.313	0.6 (0.3–1.1)	0.085
Yes		1	
Difficulty in breathing			
Yes	0.229	1.7 (1.1–2.6)	0.028
No		1	
Other morbidities			
Yes	0.125	0.7 (0.5–0.9)	0.002
No		1	
HIV exposed			
No	0.210	1.5 (1.0–2.2)	0.059
Yes		1	
Breastfeeding status			
Not breastfed	0.219	1.1 (0.7–1.6)	0.784
Not exclusively breastfed	0.253	1.1 (0.7–1.8)	0.650
Exclusive breastfed		1	
Current feeding practices			
Breastmilk	0.246	0.6 (0.4–1.0)	0.048
Formula/Mixed/Solid foods		1	
Antibiotics			
Yes	0.269	1.3 (0.8–2.2)	0.326
No		1	
Required respiratory support			
Yes	0.345	0.7 (0.3–1.3)	0.223
No		1	
(Intercept)	0.736	0.6 (0.1–2.4)	0.457

## Data Availability

The data presented in this study are available on request from the corresponding author due to ethical reasons.

## Abbreviations

The following abbreviations are used in this manuscript:

UNICEF	United Nations Children’s Fund
GBD	Global Burden of Disease
RSV	Respiratory syncytial virus
WHO	World Health Organization
NMCH	Nelson Mandela Central Hospitals
TB	Tuberculosis

## References

[1] Solomon Y., Kofole Z., Fantaye T., Ejigu S. (2022). Prevalence of pneumonia and its determinant factors among under-five children in Gamo Zone, southern Ethiopia, 2021. Front. Pediatr..

[2] Gunjak M., Morty R.E. (2022). World health day observances in November 2022: Pneumonia, chronic obstructive pulmonary disease, preterm birth, and antimicrobial resistance in focus. Am. J. Physiol.-Lung Cell. Mol. Physiol..

[3] Selvi M., Vaithilingan S. (2024). Childhood pneumonia in low-and middle-income countries: A systematic review of prevalence, risk factors, and healthcare-seeking behaviors. Cureus.

[4] Quach A., Spence H., Nguyen C., Graham S.M., von Mollendorf C., Mulholland K., Russell F.M. (2022). Slow progress towards pneumonia control for children in low-and-middle income countries as measured by pneumonia indicators: A systematic review of the literature. J. Glob. Health.

[5] Bender R.G., Sirota S.B., Swetschinski L.R., Dominguez R.M.V., Novotney A., Wool E.E., Ikuta K.S., Vongpradith A., Rogowski E.L.B., Doxey M. (2024). Global, regional, and national incidence and mortality burden of non-COVID-19 lower respiratory infections and aetiologies, 1990–2021: A systematic analysis from the Global Burden of Disease Study 2021. Lancet Infect. Dis..

[6] Perin J., Mulick A., Yeung D., Villavicencio F., Lopez G., Strong K.L., Prieto-Merino D., Cousens S., Black R.E., Liu L. (2022). Global, regional, and national causes of under-5 mortality in 2000–2019: An updated systematic analysis with implications for the Sustainable Development Goals. Lancet Child Adolesc. Health.

[7] Leung A.K., Wong A.H., Hon K.L. (2018). Community-acquired pneumonia in children. Recent Pat. Inflamm. Allergy Drug Discov..

[8] (2022). The Childhood Acute Illness and Nutrition (CHAIN) Network. Childhood mortality during and after acute illness in Africa and south Asia: A prospective cohort study. Lancet Glob. Health.

[9] Price J., Willcox M., Kabudula C.W., Herbst K., Hinton L., Kahn K., Harnden A. (2019). Care pathways during a child’s final illness in rural South Africa: Findings from a social autopsy study. PLoS ONE.

[10] Zakayo S.M., Njeru R.W., Sanga G., Kimani M.N., Charo A., Muraya K., Sarma H., Uddin F., Berkley J.A., Walson J.L. (2020). Vulnerability and agency across treatment-seeking journeys for acutely ill children: How family members navigate complex healthcare before, during and after hospitalisation in a rural Kenyan setting. Int. J. Equity Health.

[11] Izu A., Nunes M.C., Solomon F., Baillie V., Serafin N., Verwey C., Moore D.P., Laubscher M., Ncube M., Olwagen C. (2023). All-cause and pathogen-specific lower respiratory tract infection hospital admissions in children younger than 5 years during the COVID-19 pandemic (2020–2022) compared with the pre-pandemic period (2015–2019) in South Africa: An observational study. Lancet Infect. Dis..

[12] Kehoe K., Morden E., Jacobs T., Zinyakatira N., Smith M., Heekes A., Murray J., le Roux D.M., Wessels T., Richards M. (2023). Comparison of paediatric infectious disease deaths in public sector health facilities using different data sources in the Western Cape, South Africa (2007–2021). BMC Infect. Dis..

[13] Pratt M.T., Abdalla T., Richmond P.C., Moore H.C., Snelling T.L., Blyth C.C., Bhuiyan M.U. (2022). Prevalence of respiratory viruses in community-acquired pneumonia in children: A systematic review and meta-analysis. Lancet Child Adolesc. Health.

[14] Nascimento-Carvalho A.C., Vilas-Boas A.L., Fontoura M.S.H., Vuorinen T., Nascimento-Carvalho C.M., PNEUMOPAC-Efficacy Study Group (2018). Respiratory viruses among children with non-severe community-acquired pneumonia: A prospective cohort study. J. Clin. Virol..

[15] Lwanga C., Aber P., Tickell K.D., Ngari M.M., Mukisa J., Atuhairwe M., Brown L., Mupere E., Potani I., Shahrin L. (2024). Impact of HIV exposure without infection on hospital course and mortality among young children in sub-Saharan Africa: A multi-site cohort study. BMC Med..

[16] Sulley S., Ndanga M. (2019). Pediatric pneumonia: An analysis of cost & outcome influencers in the United States. Int. J. Pediatr. Adolesc. Med..

[17] Wang J., Xu Z.H., Lu J. (2022). Hospitalization costs for children with pneumonia in Shanghai, China from 2019 to 2020. Hum. Vaccines Immunother..

[18] Ilboudo A.K., Cissé A., Milucky J., Tialla D., Mirza S.A., Diallo A.O., Bicaba B.W., Charlemagne K.J., Diagbouga P.S., Owusu D. (2024). Predictors of severity and prolonged hospital stay of viral acute respiratory infections (ARI) among children under five years in Burkina Faso, 2016–2019. BMC Infect. Dis..

[19] Patel N., Ban A.S., Gladfelter T., Tripathi S. (2022). Epidemiology and outcomes of bacterial coinfection in hospitalized children with respiratory viral infections: A single center retrospective chart review. J. Pediatr. Pharmacol. Ther..

[20] Avelino I.C., Van-Dunem J., Varandas L. (2025). Under-five mortality and social determinants in Africa: A systematic review. Eur. J. Pediatr..

[21] Avelino I.C., Van-Dúnem J., Varandas L. (2024). Under-five mortality and associated risk factors in children hospitalized at David Bernardino Pediatric Hospital (DBPH), Angola: A hierarchical approach. Int. J. Environ. Res. Public Health.

[22] Barwe T.V., Mbele S.K., Ngake B.K., Tsawe M., Temane M.D., Miyambu L.N. (2024). Multilevel modelling of the determinants of under-five deaths in South Africa: Evidence from the 2016 Demographic Health Survey. Afr. J. Reprod. Health.

[23] World Health Organization (2014). Revised WHO Classification and Treatment of Pneumonia in Children at Health Facilities: Evidence Summaries.

[24] Zar H.J., Moore D.P., Andronikou S., Argent A.C., Avenant T., Cohen C., Green R.J., Itzikowitz G., Jeena P., Masekela R. (2020). Diagnosis and management of community-acquired pneumonia in children: South African Thoracic Society guidelines. Afr. J. Thorac. Crit. Care Med..

[25] Hirani R., Podder D., Stala O., Mohebpour R., Tiwari R.K., Etienne M. (2025). Strategies to reduce hospital length of stay: Evidence and challenges. Medicina.

[26] IBM Corp (2023). SPSS Statistics for Windows.

[27] Dinku H., Amare D., Mulatu S., Abate M.D. (2023). Predictors of prolonged hospitalization among children aged 2–59 months with severe community-acquired pneumonia in public hospitals of Benishangul-Gumuz Region, Ethiopia: A multicenter retrospective follow-up study. Front. Pediatr..

[28] Fernandez G.A., Vatcheva K.P. (2022). A comparison of statistical methods for modeling count data with an application to hospital length of stay. BMC Med. Res. Methodol..

[29] Wanyana M.W., Migisha R., King P., Muhesi A.K., Kwesiga B., Kadobera D., Bulage L., Ario A.R. (2024). Factors associated with severe pneumonia among children <5 years, Kasese District, Uganda: A case-control study, January–April 2023. Pneumonia.

[30] Le Roux D.M. (2024). Childhood deaths due to pneumonia: A novel causal analysis of aetiology. Lancet Child Adolesc. Health.

[31] Mahtab S., Blau D.M., Madewell Z.J., CHAMPS Consortium (2024). Post-mortem investigation of deaths due to pneumonia in children aged 1–59 months in sub-Saharan Africa and South Asia from 2016 to 2022: An observational study. Lancet Child Adolesc. Health.

[32] Owusu Konadu S., Owusu S.K., Osei-Akoto A., Nyarko O.O., Opoku G., Osei F.A. (2019). Pneumonia dominance in under-five mortalities in sub-Saharan Africa-Urgency for more research and capacity improvement. Afr. J. Curr. Med. Res..

[33] Ragwar V., Brown M. (2023). Causal factors of childhood pneumonia high mortalities and the impact of community case management on child survival in Sub-Saharan Africa: A systematic review. Public Health.

[34] Sedney C.J., Harvill E.T. (2023). The neonatal immune system and respiratory pathogens. Microorganisms.

[35] Empey K.M., Fixman E.D., Cormier S., Kolls J.K., Piedimonte G. (2023). Neonatal host immune responses to pulmonary infections. Front. Immunol..

[36] Pieren D.K.J., Boer M.C., de Wit J. (2022). The adaptive immune system in early life: The shift makes it count. Front. Immunol..

[37] Rahman A.E., Hossain A.T., Nair H., Chisti M.J., Dockrell D., El Arifeen S., Campbell H. (2022). Prevalence of hypoxaemia in children with pneumonia in low-income and middle-income countries: A systematic review and meta-analysis. Lancet Glob. Health.

[38] Restori K.H., Srinivasa B.T., Ward B.J., Fixman E.D. (2018). Neonatal immunity, respiratory virus infections, and the development of asthma. Front. Immunol..

[39] Ginsburg A.S., Mvalo T., Hwang J., Phiri M., McCollum E.D., Maliwichi M., Schmicker R., Phiri A., Lufesi N., May S. (2021). Malawian children with fast-breathing pneumonia with and without comorbidities. Pneumonia.

[40] Mwananyanda L., Thea D.M., Chipeta J., Kwenda G., Mulindwa J.M., Mwenechanya M., Wa-Somwe S., Feikin D.R., Haddix M., Hammitt L.L. (2021). The etiology of pneumonia in Zambian children: Findings from the Pneumonia Etiology Research for Child Health (PERCH) Study. Pediatr. Infect. Dis. J..

[41] Jacobs C., Nkwemu C., Ngambi B.B., Silavwe V., Qazi S.A., Nisar Y.B. (2025). Outcomes of children aged 2–59 months with chest indrawing pneumonia managed on an outpatient basis in selected primary health facilities in Zambia. J. Glob. Health.

[42] Hooli S., Makwenda C., Lufesi N., Colbourn T., Mvalo T., McCollum E.D., King C. (2023). Implication of the 2014 World Health Organization Integrated Management of Childhood Illness Pneumonia Guidelines with and without pulse oximetry use in Malawi: A retrospective cohort study. Gates Open Res..

[43] Mvalo T., Smith A.G., Eckerle M., Hosseinipour M.C., Kondowe D., Vaidya D., Liu Y., Corbett K., Nansongole D., Mtimaukanena T.A. (2022). Antibiotic treatment failure in children aged 1 to 59 months with World Health Organization-defined severe pneumonia in Malawi: A CPAP IMPACT trial secondary analysis. PLoS ONE.

[44] Mo Y., Tan W.C., Cooper B.S. (2025). Antibiotic duration for common bacterial infections—A systematic review. JAC-Antimicrob. Resist..

[45] Kato H. (2024). Antibiotic therapy for bacterial pneumonia. J. Pharm. Health Care Sci..

[46] Sithu Shein A.M., Hongsing P., Khatib A., Phattharapornjaroen P., Miyanaga K., Cui L., Shibuya K., Amarasiri M., Monk P.N., Kicic A. (2024). Phage therapy could be key to conquering persistent bacterial lung infections in children. npj Antimicrob. Resist..

[47] Le Roux D.M., Nicol M.P., Vanker A., Nduru P.M., Zar H.J. (2021). Factors associated with serious outcomes of pneumonia among children in a birth cohort in South Africa. PLoS ONE.

[48] Ndlovu S., David-Govender C., Tinarwo P., Naidoo K.L. (2022). Changing mortality amongst hospitalised children with severe Acute Malnutrition in KwaZulu-Natal, South Africa, 2009–2018. BMC Nutr..

[49] Tsegaw T.K., Alemaw H.B., Wale Y.B., Nigatu S.G., Birhan T.Y., Taddese A.A. (2024). Incomplete immunization uptake and associated factors among children aged 12–23 months in sub-Saharan African countries; multilevel analysis evidenced from latest demography and health survey data, 2023. Ital. J. Pediatr..

[50] Silaba M., Ooko M., Bottomley C., Sande J., Benamore R., Park K., Ignas J., Maitland K., Mturi N., Makumi A. (2019). Effect of 10-valent pneumococcal conjugate vaccine on the incidence of radiologically confirmed pneumonia and clinically-defined pneumonia in Kenyan children: An interrupted time-series analysis. Lancet Glob. Health.

[51] Adamu A.L., Ojal J., Abubakar I.S., Bello M.M., Odeyemi K., Okoromah C.A., Inem V., Karia B., Karani A., Akech D. (2022). Predicted serotype distribution in invasive pneumococcal disease (IPD) among children less than five years prior to the introduction of the Pneumococcal Conjugate Vaccine (PCV) in Nigeria. medRxiv.

[52] Graham H.R., King C., Duke T., Ahmed S., Baqui A.H., Colbourn T., Falade A.G., Hildenwall H., Hooli S., Kamuntu Y. (2024). Hypoxaemia and risk of death among children: Rethinking oxygen saturation, risk-stratification, and the role of pulse oximetry in primary care. Lsancet Glob. Health.

[53] Mushumbamwiza H., Webster H.H., Kayitesi C., Mill J., Chizyuka N.A., Musabirema F., Ngwije A., Kateera B., Musafiri S., Tuyisenge L. (2025). Hypoxemia detection and oxygen therapy practices in neonatal and pediatric wards across seven district and referral hospitals in Rwanda. Front. Pediatr..

[54] Schuh H.B., Hooli S., Ahmed S., King C., Roy A.D., Lufesi N., Islam A.A., Mvalo T., Chowdhury N.H., Ginsburg A.S. (2023). Clinical hypoxemia score for outpatient child pneumonia care lacking pulse oximetry in Africa and South Asia. Front. Pediatr..

[55] Li A.J., Tabu C., Shendale S., Okoth P.O., Sergon K., Maree E., Mugoya I.K., Machekanyanga Z., Onuekwusi I.U., Ogbuanu I.U. (2020). Qualitative insights into reasons for missed opportunities for vaccination in Kenyan health facilities. PLoS ONE.

[56] Agho K.E., Ezeh O.K., Ghimire P.R., Uchechukwu O.L., Stevens G.J., Tannous W.K., Fleming C., Ogbo F.A., Global Maternal and Child Health Research Collaboration (GloMACH) (2019). Exclusive breastfeeding rates and associated factors in 13 “economic community of West African states” (ECOWAS) countries. Nutrients.

[57] Zong X.N., Wu H., Zhao M., Magnussen C.G., Xi B. (2021). Global prevalence of WHO infant feeding practices in 57 LMICs in 2010–2018 and time trends since 2000 for 44 LMICs. eClinicalMedicine.

[58] North K., Gao M., Allen G., Lee A.C. (2022). Breastfeeding in a global context: Epidemiology, impact, and future directions. Clin. Ther..

[59] Adal O., Tsehay Y.T., Ayenew B., Abate T.W., Mekonnen G.B., Mulatu S., Mamo S.T., Ayenew T., Messelu M.A., Belayneh A.G. (2025). The burden and predictors of hospital-acquired infection in intensive care units across Sub-Sahara Africa: Systematic review and metanalysis. BMC Infect. Dis..

[60] Kollef M.H., Torres A., Shorr A.F., Martin-Loeches I., Micek S.T. (2021). Nosocomial infection. Crit. Care Med..

